# Supplementary material for: Serum Metabolomics Reveals a Potential Benefit of Methionine in Type 1 Diabetes Patients with Poor Glycemic Control and High Glycemic Variability
Source: Nutrients. 2023 Jan 19;15(3):518. doi: 10.3390/nu15030518 (PMC9921163; doi:10.3390/nu15030518)
Supplement: Supplementary file 1 [file nutrients-15-00518-s001.zip › nutrients-2100038-supplementary.pdf]

Supplementary materials

Table S1 Identification of differential metabolic pathways in GV-H group and GV-L group in the exploratory set

Pathway_name	Pathway_id	Total	Hits	*P* value	Impact
Linoleic acid metabolism	hsa00591	28	4	0.008473	0.1842
Aminoacyl-tRNA biosynthesis	hsa00970	52	5	0.017526	0.0982
ABC transporters	hsa02010	138	9	0.019276	0.0652
Taurine and hypotaurine metabolism	hsa00430	22	3	0.025547	0.1176
Phenylalanine metabolism	hsa00360	60	5	0.030639	0.0854
Cysteine and methionine metabolism	hsa00270	63	5	0.036812	0.2089
Alanine, aspartate and glutamate metabolism	hsa00250	28	3	0.047885	0.3153

Note: Pathway_id, ID number of target pathway in KEGG database; Total, total number of metabolites in target metabolic pathway; Hits, number of overall differential metabolites in target metabolic pathway; *P* value, *P* value of hypergeometric distribution test, the smaller *P* value, the more significant the effect of detected differential metabolites on this pathway; Impact, the effect value of metabolic pathway, indicating the effect of detected differential metabolites on the target pathway.

Table S2 Basic characteristics and CGM parameters in GV-H and GV-L groups of the validation set

	GV-H group (*n* = 11)	GV-L group (*n* = 10)	*P* value
Age (years)	26.0 (19.5, 32.0)	28.5 (16.0, 32.0)	0.805
Sex (M/F)	6/5	7/3	0.477
BMI (kg/m^2)	19.6 ± 2.2	19.3 ± 2.5	0.769
Duration (years)	1.0 (0.8, 8.4)	1.9 (0.7, 9.8)	0.944
Insulin (U/kg·d)	0.59 (0.50, 0.69)	0.60 (0.32, 0.73)	0.453
FBG (mmol/L)	8.4 ± 1.5	7.1 ± 1.4	0.055
2hBG (mmol/L)	15.1 (11.9, 20.9)	15.8 (11.1, 19.2)	0.753
HbA1c (%)	9.2 (6.3, 12.5)	7.5 (5.7, 8.3)	0.170
FCP (pmol/L)	83.2 (27.4, 164.0)	64.8 (16.5, 182.9)	0.915
2hCP (pmol/L)	122.4 (17.9, 229.0)	202.6 (24.6, 412.4)	0.321
TC (mmol/L)	3.9 ± 0.5	4.4 ± 1.0	0.218
TG (mmol/L)	0.71 (0.54, 0.89)	0.67 (0.40, 0.98)	0.972
HDL (mmol/L)	1.5 ± 0.3	1.7 ± 0.7	0.338
LDL (mmol/L)	2.1 ± 0.5	2.4 ± 0.9	0.438
SD (mmol/L)	4.2 (3.6, 4.7)	1.9 (1.4, 2.1)	**<0.001**
MAGE (mmol/L)	9.1 (8.5, 10.6)	4.2 (3.6, 4.7)	**<0.001**
CV (%)	46.2 ± 5.2	25.3 ± 6.0	**<0.001**
LBGI	3.8 (3.2, 6.6)	1.8 (1.1, 3.4)	**0.005**

Note: Data were expressed as mean ± SD, median (25th percentile, 75th percentile) and ratio. Abbreviations: BMI, body mass index; FBG, fasting blood glucose; 2hBG, 2-hour postprandial blood glucose; HbA1c, hemoglobin A1c; FCP, fasting C-peptide; 2hCP, 2-hour postprandial C-peptide; TC, total cholesterol; HDL, high density lipoprotein; LDL, low density lipoprotein; TG, triglyceride; SD, standard deviation; MAGE, mean amplitude of glucose excursions; CV, coefficient of variation; LBGI, low blood glucose index.

Table S3 Performance of candidate metabolic markers in the validation set

Metabolite	FC in the exploratory set	FC in the validation set	*P* value
Phosphatidylcholine	119.49	0.01	<0.001
3-(2-Hydroxyphenyl)propanoic acid	0.47	0.71	0.231
Riboflavin	0.51	2.11	<0.001
9,10-DHOME	0.22	5.70	<0.001
Cysteine-S-sulfate	0.16	8.70	0.002
Phenyllactate	0.48	1.60	0.360
13S-hydroxyoctadecadienoic acid	0.53	1.10	0.526
L-Cysteine	0.23	6.53	0.001
**Spermidine**	0.56	0.55	**0.007**
3-Methylthiopropionic acid	0.43	1.52	0.260
Hydrocinnamic acid	0.53	2.90	0.024
**Trehalose**	3.33	1.49	**0.041**
D-Phenylalanine	1.31	1.09	0.622
Oxoglutaric acid	1.56	0.72	0.324
2-Ketobutyric acid	1.28	0.90	0.360
Phenylethylamine	0.12	44.0	<0.001
Phutalic acid	0.02	4.66	0.001
12,13-DHOME	0.02	53.6	<0.001
D-Xylitol	2.55	0.84	0.324
**L-Methionine**	1.44	1.23	**0.041**
N-Acetyl-L-aspartic acid	1.21	0.69	0.014
L-Glutamic acid	1.28	0.70	0.833
L-Histidine	1.29	1.10	0.725
Mannitol	2.92	0.49	0.020
L-Valine	1.3	0.92	0.360

Note: *P* value represents the result of hypergeometric distribution test in the validation set.

Figure S1

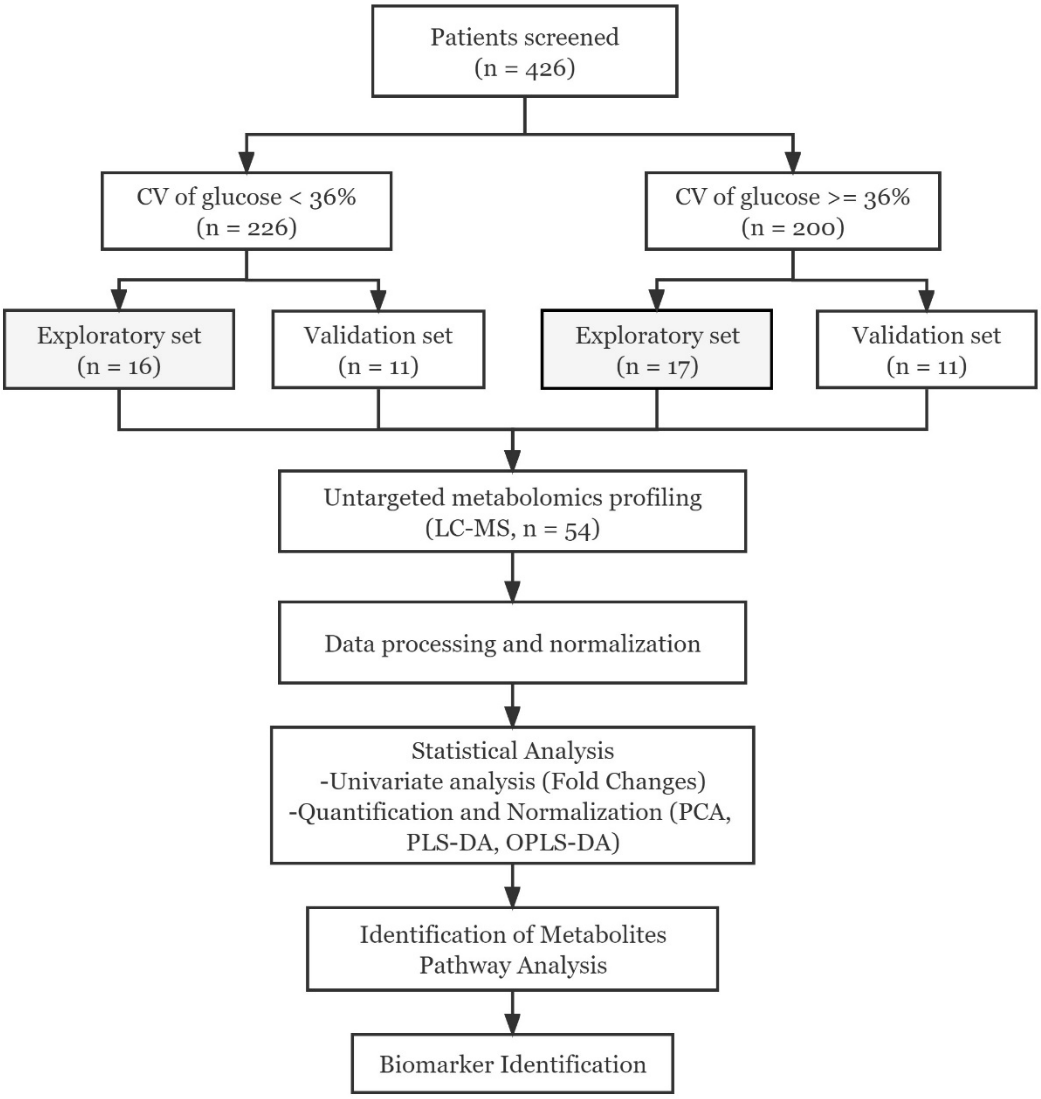

Figure S1. Flow chart of the comprehensive analysis of metabolomics profiles of patients with different glycemic variability

Table S4 Basic characteristics and CGM parameters of patients enrolled and not enrolled

	Enrolled (n = 54)	Not enrolled (n = 372)	P value
Age (years)	25.0 (15.0, 34.0)	22.0 (14.0, 35.0)	0.245
Sex (M/F)	32/22	190/182	0.167
BMI (kg/m^2)	19.8 ± 2.5	20.1 ± 3.3	0.140
Duration (years)	1.2 (0.7, 6.2)	1.8 (0.4, 4.7)	0.505
Insulin (U/kg·d)	0.62 (0.48, 0.74)	0.61 (0.45, 0.76)	0.875
FBG (mmol/L)	8.1 (6.9, 10.1)	8.0 (5.4, 10.8)	0.748
2hBG (mmol/L)	16.6 (13.7 19.7)	14.3 (11.1, 18.7)	0.026
HbA1c (%)	7.9 (6.8, 9.3)	7.6 (6.7, 9.2)	0.691
FCP (pmol/L)	55.5 (16.8, 118.9)	60.8 (16.5, 138.5)	0.081
2hCP (pmol/L)	122.2 (21.9, 228.8)	100.3 (25.7, 295.3)	0.766
TC (mmol/L)	4.2 ± 0.7	4.3 ± 1.0	0.485
TG (mmol/L)	0.69 (0.53, 0.88)	0.75 (0.56, 1.07)	0.247
HDL (mmol/L)	1.5 ± 0.5	1.5 ± 0.4	0.734
LDL (mmol/L)	2.4 ± 0.6	2.5 ± 0.9	0.715
SD (mmol/L)	3.2 (2.4, 3.9)	3.0 (2.5, 3.8)	0.746
MAGE (mmol/L)	7.0 (4.9, 8.8)	6.8 (5.2, 8.4)	0.380
CV (%)	32.3 (26.2, 45.4)	34.9 (30.0, 39.5)	0.505
LBGI	3.2 (1.3, 4.8)	2.9 (1.8, 4.5)	0.617
